# A Review of Sustained Drug Release Studies from Nanofiber Hydrogels

**DOI:** 10.3390/biomedicines9111612

**Published:** 2021-11-04

**Authors:** Ilker S. Bayer

**Affiliations:** Smart Materials, Istituto Italiano di Tecnologia, 16163 Genova, Italy; ilker.bayer@iit.it; Tel.: +39-380-387-6699

**Keywords:** nanofiber, hydrogel, nanofiber hydrogel, drug release, gel rheology

## Abstract

Polymer nanofibers have exceptionally high surface area. This is advantageous compared to bulk polymeric structures, as nanofibrils increase the area over which materials can be transported into and out of a system, via diffusion and active transport. On the other hand, since hydrogels possess a degree of flexibility very similar to natural tissue, due to their significant water content, hydrogels made from natural or biodegradable macromolecular systems can even be injectable into the human body. Due to unique interactions with water, hydrogel transport properties can be easily modified and tailored. As a result, combining nanofibers with hydrogels would truly advance biomedical applications of hydrogels, particularly in the area of sustained drug delivery. In fact, certain nanofiber networks can be transformed into hydrogels directly without the need for a hydrogel enclosure. This review discusses recent advances in the fabrication and application of biomedical nanofiber hydrogels with a strong emphasis on drug release. Most of the drug release studies and recent advances have so far focused on self-gelling nanofiber systems made from peptides or other natural proteins loaded with cancer drugs. Secondly, polysaccharide nanofiber hydrogels are being investigated, and thirdly, electrospun biodegradable polymer networks embedded in polysaccharide-based hydrogels are becoming increasingly popular. This review shows that a major outcome from these works is that nanofiber hydrogels can maintain drug release rates exceeding a few days, even extending into months, which is an extremely difficult task to achieve without the nanofiber texture. This review also demonstrates that some publications still lack careful rheological studies on nanofiber hydrogels; however, rheological properties of hydrogels can influence cell function, mechano-transduction, and cellular interactions such as growth, migration, adhesion, proliferation, differentiation, and morphology. Nanofiber hydrogel rheology becomes even more critical for 3D or 4D printable systems that should maintain sustained drug delivery rates.

## 1. Introduction

### Definition of Hydrogels and Their Applications

**Figure 1:**
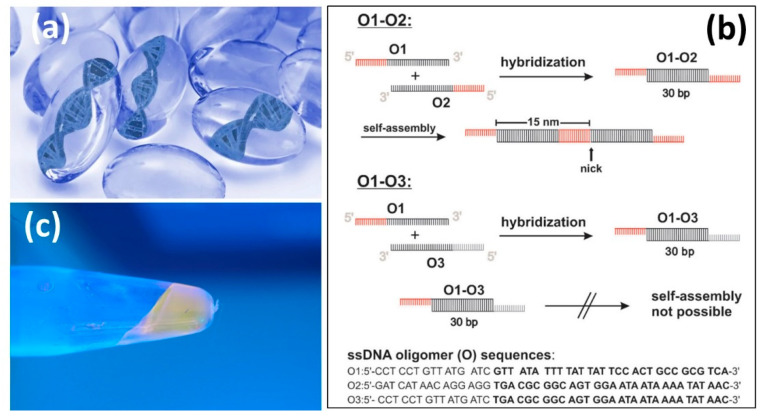
(**a**) DNA based hydrogels. DNA can be used as the sole component of a hydrogel. It can act as the backbone or a cross-linking agent connecting active molecules to the hydrogel via chemical reactions or physical entanglement. (**b**) A typical DNA hydrogel formation mechanism known as oligomer hybridization. By hybridization of O1 with O2, a double-stranded DNA monomer (O1-O2, 30 base pairs and 15 base sticky ends) is generated, which can self-assemble. The double-stranded DNA monomer O1-O3 (30 base pairs) comprises non-complementary 15-base overhangs and cannot further self-assemble. (**c**) Optical micrograph of a DNA hydrogel produced from the oligomers O1 and O2. The DNA hydrogel was stained with CybrSafe^®^ DNA dye and illuminated with UV light (λmax = 366 nm). Reproduced from [2,6] with permission.

A hydrogel is defined as a three-dimensional (3D) network of hydrophilic molecules or polymers that are able to swell in water and retain large amounts of water in their bulk. In such a state, hydrogels can maintain their structure and stability due to chemical or physical cross-linking of individual polymer chains. The first reported hydrogels in the literature were due to Wichterle and Lím (1960) [1]. It is generally accepted that water must constitute at least 10% of the total weight or volume of the polymeric network to be labeled as a hydrogel. Hydrogels should feature a good degree of flexibility identical to natural tissue. The hydrophilicity of the polymer-water network is maintained by the presence of hydrophilic groups such as -NH_2_, -COOH, -OH, -CONH_2_, -CONH-, and -SO_3_H. Hydrogel technologies are rapidly evolving, and new examples are emerging, such as DNA hydrogels (see Figure 1) that are known as swollen networks of DNA molecules cross-linked in an aqueous solution [2,3,4]. DNA is a biological polymer that is hydrophilic, biocompatible, and highly programmable by Watson–Crick base pairing (i.e., adenine (A) forms a base pair with thymine (T) using two hydrogen bonds, and guanine (G) forms a base pair with cytosine (C) using three hydrogen bonds). DNA can be transformed into a hydrogel by itself under certain conditions, and it can also be integrated into synthetic polymers to form DNA-hybrid hydrogels. Functional DNAs, such as aptamers and DNAzymes, offer additional molecular recognition capabilities and versatility [5].

In Figure 1b,c, a schematic and a photograph show a three-dimensional DNA hydrogel that was made by self-assembly of short linear double-stranded DNA building blocks furnished with sticky ends. The resulting DNA hydrogel was thermo-responsive, and the length of the supramolecular double-stranded DNA structures varied with temperature [6]. Proper selection of oligomer pairs is critical to ensure the formation of hydrogels; for instance, pairing **O1** and **O3** oligomers does not result in self-assembly and gelation (Figure 1b). A recent review [7] studied the existing data and methods related to the mechanical design of pristine DNA and hybrid hydrogels and their use in cell culture. The focus of the article was to advance further studies toward designing multifunctional DNA hydrogels with programmable and controlled mechanical properties.

In medicine, hydrogels are used as scaffolds for tissue engineering, sustained drug release media, optical and microfluidic actuation, and as model extracellular matrices in biotechnology. Applications of hydrogels can be limited by their mechanical response. Most hydrogels are not elastomeric networks with low stretching, such as an alginate hydrogel that ruptures upon elongation to about 1.2 times its original length [8]. Most hydrogels are still classified as brittle, with fracture energies of about 10 Jm^−2^ compared to ∼1000 Jm^−2^ for cartilage and ∼10,000 Jm^−2^ for natural rubbers [8].

**Figure 2:**
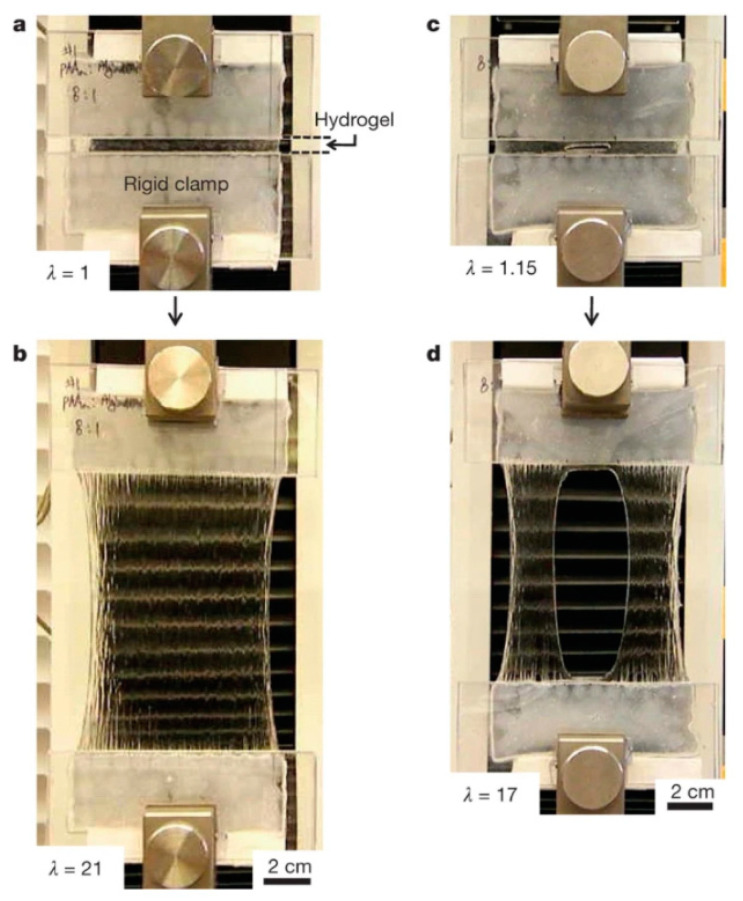
(**a**) The non-perturbed hydrogel between two rigid clamps. (**b**) The gel was stretched 21 times its initial length in a tensile machine. (**c**) The gel was damaged by forming razor blade notch. (**d**) Due to the damage, the stretching was 17 times the initial length instead, but still significant. Reproduced from [8] with permission.

The flexibility and stretching and elastic recovery of hydrogels are very important sought-after properties, as shown in Figure 2, because poor mechanical stability of hydrogels used for cell encapsulation can cause involuntary cell release and death [9], and low toughness severely restricts the durability of contact lenses [10]. In Figure 2, a highly stretchable, tough hydrogel is demonstrated by mixing two types of cross-linked polymers, namely, ionic cross-linking of alginates and covalently cross-linked polyacrylamide [8].

**Figure 3:**
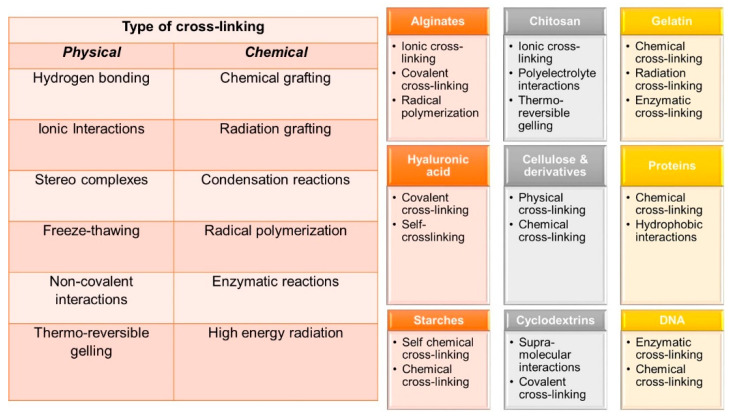
Typical hydrogel forming interactions and cross-linking pathways. The figure also displays widely used hydrogel-forming reactions of natural polymers including DNA.

As mentioned earlier, hydrogels are three-dimensional networks in which hydrophilic polymers cross-link together. Figure 3 summarizes typical physical and chemical cross-linking reactions leading to hydrogels, as well as a list of common natural polymers and relevant cross-linking pathways used in transforming them into hydrogels.

**Figure 4:**
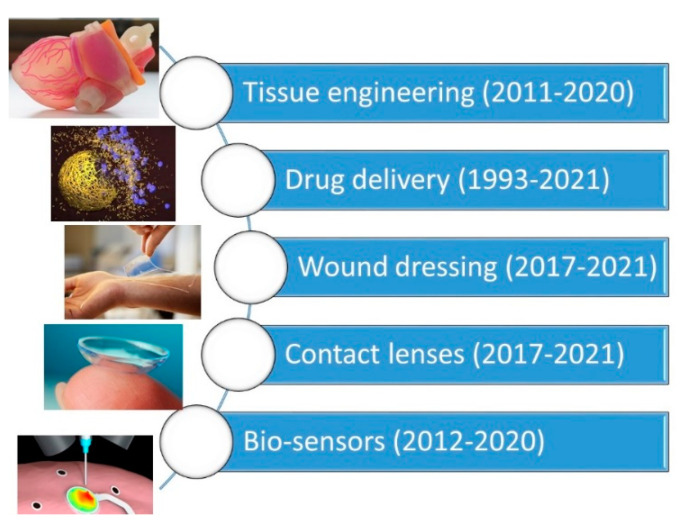
Schematic display of common hydrogel applications and the publication years of comprehensive reviews relevant to each application.

There are several dedicated reviews on each of these natural polymer based hydrogels and more and the readers should consult to them for further details on cross-linking and applications. For instance, alginate based hydrogels have been reviewed and discussed in [11,12,13,14,15]. Chitosan based hydrogels have been presented and discussed in [16,17,18,19,20]. Gelatin and protein based hydrogels have been extensively studied and classified in [21,22,23,24,25,26,27,28,29,30]. Cellulose based hydrogels are also very common and reviewed several times in the literature with relevant applications [31,32,33,34,35,36,37,38]. Similarly, starch based hydrogels are also common and have been analyzed carefully in [39,40,41,42,43,44,45,46]. Cyclodextrins are a family of cyclic oligosaccharides, consisting of a macrocyclic ring of glucose subunits joined by α-1,4 glycosidic bonds. Cyclodextrins are produced from starch by enzymatic conversion. They are used in food, pharmaceutical, drug delivery, and chemical industries, as well as agriculture and environmental engineering. Hydrogels based on cyclodextrins are extensively reviewed in [47,48,49,50,51,52,53]. DNA based hydrogels are rapidly gaining in popularity and can find a wide range of applications in the biomedical field. Hyaluronic acid (HA), also called hyaluronan, is an anionic, non-sulfated glycosaminoglycan distributed widely throughout connective, epithelial, and neural tissues. It is unique among glycosaminoglycans as it is non-sulfated, forms in the plasma membrane, and can have very high molecular weight; for instance, human synovial HA averages about 7 million Da per molecule, or about 20,000 disaccharide monomers, and some HA molecules extracted from tissues can reach 3–4 million Da [54]. HA based hydrogels are extremely biocompatible materials that can be rendered functional by various approaches including cross-linking in the presence of drugs and particularly for cancer treatment [55,56,57,58,59,60,61]. Chemical and physical properties of various DNA based hydrogels as well as their properties can be found in [62,63,64,65,66,67,68]. There is even a much larger body of literature on hydrogels based solely on synthetic polymers and/or hydrogel interpenetrating networks between natural and synthetic polymers, as well as their successful applications, and readers can refer to a number of reviews in [67,68,69,70,71,72,73,74,75,76,77,78,79]. Figure 4 shows typical general applications of hydrogels in the biomedical field, and the figure shows a timeline of review articles that have been published on each application field. Table 1 further lists those specific review articles that readers can refer to for the advances made in those specific periods. In particular, it can be seen that drug delivery from hydrogels is the oldest subject for review articles; however, there appear to be significant differences in the total number of reviews published on each application (Table 1). Since the general principles and applications of hydrogels are beyond the scope of this review article, readers can refer to a number of works cited herein [80]. In the following sections, we will discuss the process of electrospinning briefly and review recent advances in hydrogels made from electrospun materials while specifically focusing on controlled drug-release works from such materials.

## 2. Principles of Electrospinning, and Hydrogels from Electrospun Materials

**Figure 5:**
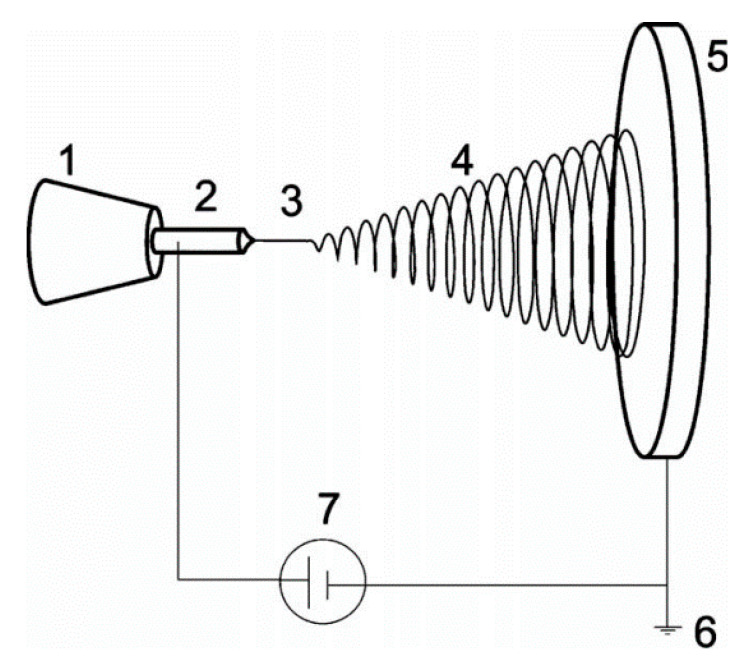
Schematic representation of electrospinning system: (**1**) syringe and metering pump, (**2**) needle/capillary acting as the electrode, (**3**) stable part of the jet, (**4**) whipping/coiling zone, (**5**) collector, (**6**) ground, and (**7**) high voltage supply. Reproduced from [112] with permission.

The technology of electrospinning is rapidly evolving, as it is a unique way to produce polymers, membranes, textiles, composites, and hydrogels based on hierarchically structured fibers with diameters typically being hundreds of nanometers [109]. The electrospinning process encompasses a complex combination of fluid mechanics, polymer science, and electrostatics. Electrostatic aerosol spraying of organic monomers can be considered as the launching pad for electrospinning and has been studied for a long time. In contrast, electrospinning applies to macromolecules or sol-gels rather than small molecules or monomers. It is generally argued that the morphology and physical entanglement of macromolecular chains can avert the capillary breakup of the electrospinning jet and result in the formation of nanofibers (Figure 5). In fact, electrospinning has been known for almost a century; with the literature including reports dating back even to the 18th century [110]. However, a real boost in electrospinning came about due to the development of nanoscience and nanotechnology [111]. Architecturally, an electrospun polymeric jet resembles a tree, as shown in Figure 5. The “roots” evolve from an exceedingly thin charged surface layer, called Debye’s layer, of the polymer solution that acts as one of the electrodes that is connected to a high voltage supply. Further downstream, a stable part of the jet is formed that looks like a tree stem. The following, larger section is the whipping zone or bending instability within the jet that resembles the branches [112].

**Figure 6:**
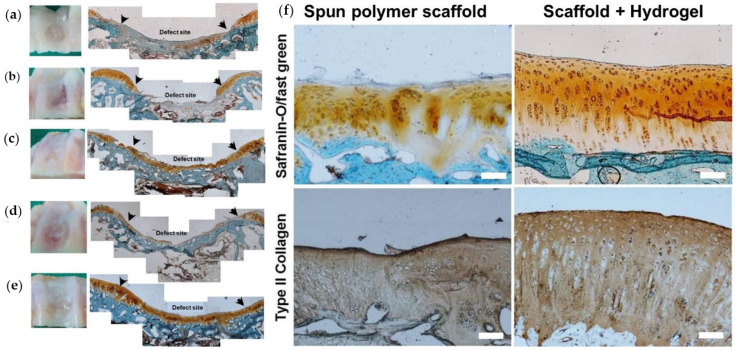
Photographs of the defect site and histological staining (safranin-O/fast green) of cryo-sectioned samples in rabbit knee at 4 months. (**a**) Control partial defect site; (**b**) electrospun polymer scaffold with no cell impregnation; (**c**) the same scaffold with cells; (**d**) the polymer scaffold/hydrogel without cells; and (**e**) the scaffold–hydrogel composite with cells. The arrows point to the borders of initial defect site. Finally, (**f**) photographs of histological (safranin-O/fast green) and immune-histochemical (type II collagen) stained images of cryo-sectioned samples in rabbit knee at 4 month after implantation of electrospun scaffolds or scaffold–hydrogel composites are shown with scale bars: 100 μm. Reproduced from [114] with permission.

Electrospun fiber networks can be transformed directly into hydrogels based on the type of polymer or polymers used, but also fiber networks can be combined with hydrogels, as in a composite, to design new functional biomedical materials [113]. In fact, hydrogel-electrospun fiber composites fortified with cells appear to be quite promising candidates for cartilage repair. In Figure 6, for instance, rabbit cartridge damage is shown to be repaired very effectively using a hydrogel-nanofiber composite. Specifically, the nanofiber hydrogel was used to deliver chondrocytes to promote the cartilage repair [114]. Self-assembled nanofiber hydrogels are considered to be utilized with collagen-like functions towards, for instance, cardiomyocyte culture in 2D and 3D [115]. Recent works showed that hydrophilic self-assembling nanofiber hydrogels can support the culture of both rat cardiomyocytes and human embryonic stem-cell-derived cardiomyocytes, which could lead to promising applications in cardiac tissue engineering [115]. Hydrogels were also fabricated from silk nanofibers by combining β-sheet-rich silk nanofibers with amorphous silk nanofibers. The composite nanofiber networks were transformed into hydrogels by horseradish peroxidase cross-linking in an electric field. Such nanofiber hydrogels demonstrated osteogenic differentiation and the aligned aggregation of stem cells in vitro, while also exhibiting osteoinductive capacity in vivo. Such improved tissue performance with nanofiber hydrogels supports encouraging applications in bone tissue engineering [116,117].

**Figure 7:**
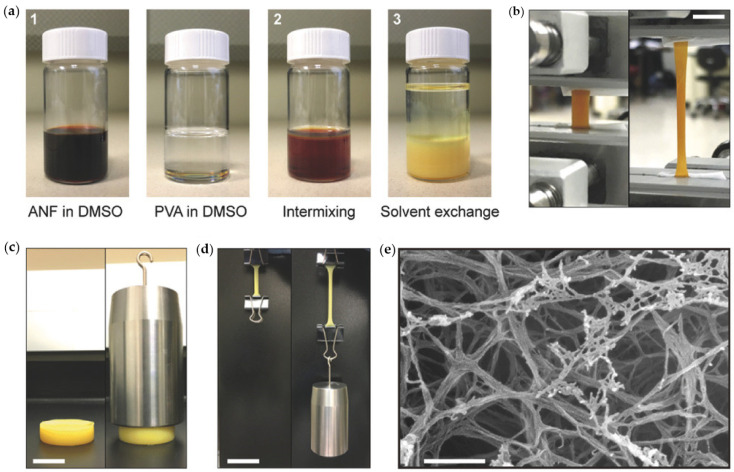
Nanofiber stretchable hydrogels made from aramid nanofibers and polyvinyl alcohol polymer. (**a**) Photographs of the polymer solutions and their mixing before electrospinning. (**b**) A hydrogel under tension with 0% (left) and 300% (right) tensile strains. Scale bar: 10 mm. (**c**) A hydrogel containing much less polyvinyl alcohol with (right) and without (left) a compressive load of 10 N. Scale bar: 30 mm. (**d**) The same sample in (**c**) with (right) and without (left) a tensile load of 10 N. Scale bar: 50 mm. (**e**) An SEM image of the same sample in (**c**) after drying. Scale bar: 300 nm. Reproduced from [115] with permission.

Nanofiber hydrogels also can be constructed from special blends of polymers that are usually immiscible with very diverse physical and chemical properties, as shown in Figure 7. Therein, polyimide and polyvinyl alcohol polymers were blended in solution and electrospun into hydrogels that demonstrate unique mechanical properties, particularly stretching ability suitable for abiotic soft tissue applications. Finally, we will discuss direct electrospinning of functional hydrogels into nanofiber networks. Direct hydrogel electrospinning is still quite challenging to be adapted to any hydrogel material system. However, several promising works have been reported, and this specific technology should be furthered possibly by utilizing the concepts of reactive electrospinning [118,119]. Very recent studies showed that co-electrospinning of biopolymers with gels or hydrogels is also possible, and that such products could be designed specifically for drug delivery [120]. For instance, polycaprolactone nanofibers were produced by co-electrospinning with vancomycin hydrochloride and simvastatin drugs that were already in gel form. The resultant hydrogel/polymer nanofiber composite could sustain drug release for as long as 14 days [120]. Another notable work [121] demonstrated synthesis of reactive macromers that contained protease-cleavable and fluorescent peptides that could form electrospun fibrous hydrogels through a photoinitiated polymerization. These nanofiber hydrogels could release hyaluronic acid for a period of 24 days near protease implants, ensuring effective implant-tissue interaction.

The technology and applications of encapsulated electrospun nanofibers in hydrogel matrices have been reviewed by Bosworth et al. [122], including the formation of coatings from such composites. Impressive sustained drug release profiles (40 days) were reported from ultraviolet light-assisted electrospinning of core–shell fully cross-linked poly(N-isopropylacrylamide-co-N-isopropylmethacrylamide) hydrogel-based nanofibers for thermally induced drug delivery [123]. Therein, a model drug (organic dye), Rhodamine B, was used instead of a medicinal compound for temperature-induced drug release. Likewise, Nakielski et al. [124] fabricated a poly(l-lactide)-Rhodamine B-loaded nanofibrous material, which encapsulated poly(Nisopropylacrylamideco-N-isopropylmethacrylamide) hydrogel containing gold nanorods that the authors indicated had resembled natural structures such as jellyfish or hydra. This nanofiber hydrogel could regulate drug release when subjected to near-infrared light stimulation. Remarkable sustained drug release profiles were obtained extending into 75 days. Furthermore, a key review article discussed diverse fabrication methods and extensive biomedical applications of nanofiber-based hydrogels ranging from bone tissue generation to sensing [125].

Table 2 summarizes recent advances in hydrogel nanofibers in terms of the types of polymer(s) used as well as resulting mechanical properties and the targeted applications. Inspection of Table 2 indicates that in a majority of these recent works, the required application target was successfully achieved without the inclusion of medicinal or other active ingredients. Only a small percentage of the studies did not measure and discuss the mechanical properties of the nanofiber hydrogels. However, careful studies on the mechanical properties of nanofiber hydrogels must be conducted regardless of the biomedical focus, particularly for cartilage repair but also for soft tissue engineering.

Special attention should be devoted to poly(N-isopropyl acrylamide) and its copolymers, since they can form a variety of thermo-responsive hydrogels but can also be electrospun into nanostructured systems [126,127]. Nanofiber/hydrogel composites based on poly(N-isopropyl acrylamide) composites also have been fabricated recently [128]. Combining cellulose nanofibers with poly(N-isopropyl acrylamide) produced enhanced swelling and compressive properties compared to pure poly(N-isopropyl acrylamide) hydrogels [128]. In fact, a unique dual-responsive (pH and temperature) composite hydrogel based on cellulose nanofibril and poly (N-isopropyl acrylamide) was developed for model drug release [129]. A model drug (methylene blue) was released from these nanofiber hydrogels for up to 2 days, triggered by either pH or temperature. Another notable work was based on the development of chitinous nanofiber-based poly(N-isopropyl acrylamide) flexible composite hydrogels for controlling cell adhesion and detachment [130]. Using these types of thermo-responsive stretchable nanofiber hydrogels, the authors could control the growth direction of the cells [130].

As mentioned at the beginning of this review, increasing the surface area of hydrogels with nanofiber networks will have tremendous implications for biomedical applications, particularly for delivering special bioactive macromolecules or drugs in a sustained manner but also facilitating newly emerging nanotechnology-mediated RNA therapies [146].

## 3. Rheology of Nanofiber Hydrogels

The rheological properties of hydrogels are strongly dependent on the molecular structure of polymeric randomly cross-linked or supramolecular gel networks. Rheological studies of hydrogels in general are important in order to identify limits of their application range. Even though the assessment of the network construction of randomly cross-linked and supramolecular gels seems to be straightforward, there are several variables that affect the gel consistency and response to stimuli through physical and chemical reactions formed by the fractal nature of the bonding. A poor understanding of the rheological properties of hydrogels can cause significant problems for practical applications. For instance, the rheological properties of peptide-based hydrogels for biomedical applications have been extensively reviewed in [147]. To clarify this point, namely the general influence of hydrogel rheological properties on cell function, we can cite a number of notable works that specifically studied these interactions. For instance, extracellular matrix (ECM) hydrogels stimulate constructive tissue remodeling in various tissues. Minimally invasive delivery of such hydrogels to the central nervous system (CNS) must be conducted by injecting a liquid form of the gel at room temperature, while forming hydrogels at body temperature. This liquid–hydrogel transformation is strongly related to the rheological properties of the system and directly impacts tissue cell response at the cavity where injection is introduced [148]. More specifically, the work of Massensini et al. [148] proved that by studying and tuning the rheology (gelation parameters) of biodegradable hydrogels, optimum cell infiltration responses for the injected hydrogels were obtained. Similarly, Du et al. [149] studied the effect of rheological properties on cell growth in peptide-based multicomponent hydrogels.

They concluded that by studying the rheology of supramolecular hydrogels, one can tune the ability of the hydrogel to efficiently store the work of deformation during cell division and transform the cell-laden hydrogel into healthy tissue. Finally, other studies showed that modulus-regulated 3D-cell proliferation in an injectable self-healing hydrogel could lead to optimized cell response and higher therapeutic efficiency for cell therapy [150].

In this section, we reviewed rheological properties of peptide and polypeptide-based hydrogels by summarizing bulk mechanical properties, gelation mechanisms, and the behavior of hydrogels during and after flow. Although these rheological results alone can be informative and, with further modeling, yield a rational understanding, rheological studies should always be considered in conjunction with structural characterization data (e.g., microscopy and scattering) for a better understanding of the observed gel properties. To that end, the authors of the aforementioned studies reviewed rheological properties of peptide and polypeptide-based hydrogels by discussing bulk mechanical properties, gelation mechanisms, and hydrogel behavior during and after flow. They suggested that anytime a new hydrogel is constructed, detailed rheological studies should be accompanied by structural characterization data (e.g., microscopy and scattering) to achieve the best biomedical application outcome.

**Figure 8:**
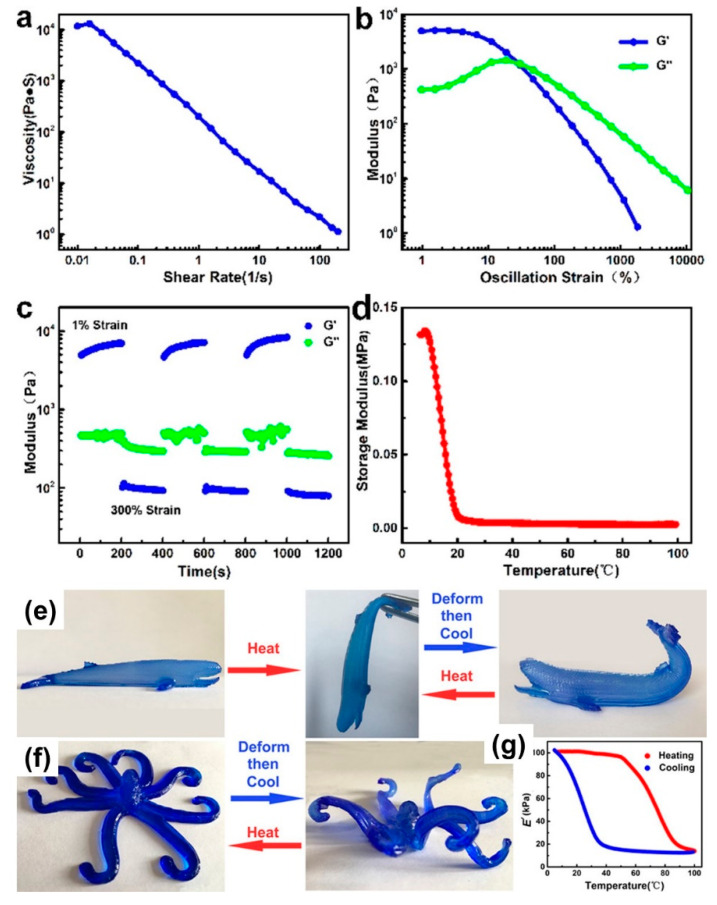
Rheological behaviors of the agarose/AM/Laponite 4D ink at 95 °C. Flow rheology pattern showing viscosity against shear rate (**a**). Oscillatory rheology pattern showing shear storage modulus (G′) and shear loss modulus (G″) evolution of inks used, increasing shear strain (**b**). Oscillatory rheology pattern showing modulus evolution of inks between 1% and 300% strain (**c**). The storage modulus variation of 4D ink during cooling (**d**). Four-dimensional printing product printed by 4D ink: a whale-like hydrogel was used as a model schematic for the 4D transition process (**e**), the softening and hardening cycles of an octopus-like gel (**f**), the storage modulus (**e**) variation of 4D gel during heating and cooling cycles measured by rheology (**g**). Reproduced from [152] with permission.

As indicated, both the flow properties of the solution and mechanical properties of the gels are critical for successful applications. Nevertheless, both properties depend on the polymer concentration, and solely increasing the concentration to improve the gel properties often leads to excessively high fluid viscosities. Thus, it was argued that hydrogels with a mixture of high molecular weight polymer and a lower molecular weight polymer would ensure decoupling the dependence of the two properties (viscosity and mechanical) from the overall concentration [151]. For instance, in the case of 3D or 4D printing of nanofiber gels, optimization of the rheological properties of the matrix is critical for high-fidelity matrix-assisted 3D printing that enables the free-form fabrication of fluidic soft materials. In Figure 8, for instance, to have a printable ink, Laponite (a type of clay) was used to induce shear-thinning behavior. As shown in Figure 8a, the rheological performance at 95 °C of the ink displayed a high viscosity (η ≈ 104 Pa·s) at low shear rate (0.01 s^−1^), while the viscosity reduced rapidly with an increase in shear rate. When the shear rate was 100 s^−1^, the viscosity was only about 1 Pa·s. The high viscosity ensures good shape stability after printing, whereas the low viscosity enables the ink to be extruded easily. The strain amplitude sweep of the ink is shown in Figure 8b, in which the storage modulus (G′) was about 5 kPa. It surpassed the loss modulus (G″) at oscillation strain below 30%, demonstrating solid-like behavior up to this strain level; at strain levels higher than 30%, G″ exceeded G′, and the ink changed from a solid-like state to a liquid-like state. Conducting continuous step strain measurements on the nanofiber hydrogels indicated that the ink was in solid state at 1% oscillation strain; after the oscillation strain was raised to 300%, it changed rapidly to a liquid state, and hence, the sol–gel transition time was sufficiently short (Figure 8c). This fast and repeatable transformation ensures good 3D or 4D printing of nanofiber hydrogels [152]. These sol–gel and gel–sol transformations of 4D ink were repeatable. Additionally, as shown in Figure 8d–g, the hydrogel ink could be solidified rapidly at ambient temperature after printing in a repeatable manner.

An optimum thermo-rheological characterization of different nanofiber hydrogels used in 3D printing is crucial to identify the best processing conditions and to extend the understanding framework to 4D printing for biomedicine. Consequently, the rheological features of printable nanofiber hydrogels, as summarized in Table 3, must be carefully monitored during processing. Although some publications demonstrated good printability of injection properties, some failed to report full thermo-rheological characteristics, as shown in Table 3. It should be noted that the nanofiber hydrogels should possess shear-thinning properties, allowing smooth extrusion from the nozzle under shear, and the extruded hydrogel must be strong enough to maintain its shape and withstand the weight of additional layers that need to be deposited.

As we summarized in this section, improved understanding and reporting of rheological properties will also translate into better theoretical explanations of how the gel components affect rheological performance along with better prediction and design of the next generation of printable biomedical nanofiber hydrogels or nanofiber/hydrogel composites [168].

## 4. Advances in Sustained Drug Release from Nanofiber Hydrogels

### 4.1. A Brief Look at Controlled Drug Release

**Figure 9:**
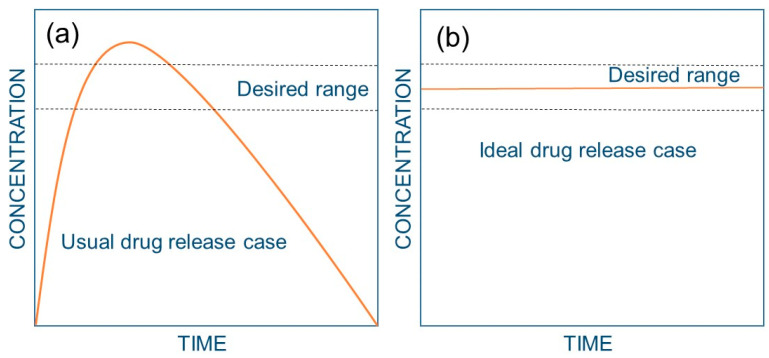
Plasma drug levels as a function of time for the usual and ideal case for drug administration. (**a**) Usual case; i.e., injection or pill: the plasma drug level reaches a peak as the drug enters the bloodstream. However, the drug quickly diminishes and falls to a low or non-existent level, dictating repeated drug administration to enable re-entry into the therapeutic range, resulting in a saw-tooth pattern of plasma drug levels. When the drug is above the desired range, it may cause unwanted side effects; when the drug is below the desired range, it no longer has a therapeutic effect. (**b**) Ideal case: a controlled-release system causes the drug level to be within the desired range for specified periods (days or months).

In order to have an efficient therapeutic effect, a drug should have high affinity and selectivity for the intended biological function. For instance, pure proteins or protein complexes should target certain cells, penetrate the membrane, and reach a sufficient concentration at that specific site. To achieve this, the drug needs to be released from the delivery system, transported from the site of application to the site of action, take part in biological reactions, and finally be eliminated via metabolic pathways. It is now generally accepted that rapidly released drugs, whether taken orally or administered in other ways, result in peaks and valleys in drug levels (Figure 9a), which may cause inefficient drug administration and, possibly, undesirable side effects [169]. Sustained drug delivery technology signifies one of the most rapidly evolving areas in human health care. These delivery systems (Figure 9b) have numerous advantages compared to conventional dosage forms, including better efficacy, reduced toxicity, and improved patient compliance and convenience. Controlled release often requires synthetic polymers as carriers for the drugs some of which were cited in [170]. A comprehensive study of drug release from hydrogels in general was conducted by Lee and Kim [171] in 1991. Therein, they conducted detailed studies on the sustained release of both hydrophilic and lipophilic model drugs. An earlier study by Lee [172] presented a detailed analysis of water-soluble drugs from initially dehydrated hydrogel matrices that generally involved the simultaneous absorption of water and desorption of drug via a swelling-controlled diffusion mechanism. The author developed a method to model constant-rate drug delivery from glassy hydrogel matrices via an immobilized non-uniform drug concentration distribution.

Very recently, a new mathematical approach to predict the actual drug release from hydrogels was published [173], taking into account hydrogel swelling and drug diffusion within the hydrogel before release. An exclusive review article on pulsed and/or pulsatile release from hydrogels was published by Kikuchi and Okano [174]. The authors argued that hydrogels could be the ideal media for developing new drug delivery devices to achieve pulsed delivery of a certain amount of a drug in order to mimic the function of living systems while minimizing undesired side effects.

Table 4 lists a number of studies in which various stimuli were utilized. The table also shows a number of review articles that specifically focused on light, pH, and electro-induced drug release from hydrogels. In many cases listed in the table, cancer drugs such as doxorubicin (chemotherapy) were extensively used as model drugs.

As can be seen in Table 4, drug release from hydrogels is generally triggered by various stimuli such as pH, magnetic fields, electricity, and light. Some of these stimuli, such as light and magnetic triggering, were also used together or in tandem. Some of the studies presented in Table 4 did not use or include any model drugs but investigated the response of the hydrogel to external stimuli in terms of changes in their viscoelastic properties.

### 4.2. Sustained Drug Release from Nanofiber Hydrogels

Nanofiber hydrogels can be fabricated by using various approaches, such as electrospinning certain polymers and swelling them in water under a controlled environment [198], embedding nanofibrillar materials (either natural or synthesized, including electrospinning) into hydrogels made by common procedures [199], or swelling nanofiber networks of natural polymers such as cellulose and chitin or chitosan in certain coagulation systems [200]. A recent review article primarily focused on recent advances in polyvinyl alcohol-polysaccharide based hydrogels and electrospun nanofibers in biomedical applications [201]. Therein, the authors argued that these systems can meet many requirements of wound management membranes in terms of surface area to volume ratio, high porosity, tolerable permeability, wound-exudates absorption capacity, architecture similarity with the extracellular matrix, and sustained release capabilities. They argued that several limitations related to scaling up electrospinning to industrial production levels and issues related to nanofiber quality reproducibility will still need attention before significant commercialization of nanofiber hydrogels made from polyvinyl alcohol and polysaccharide combinations.

**Figure 10:**
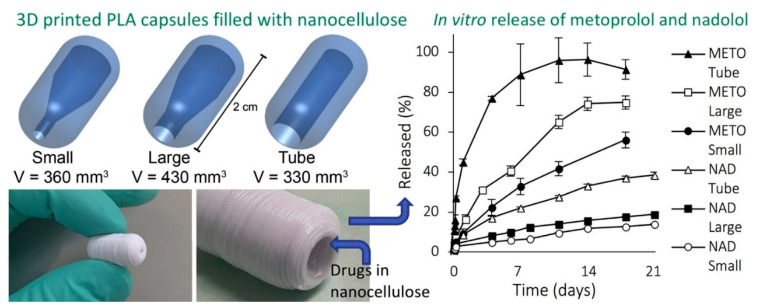
Computer-aided designs and 3D printed PLA capsules (**left**). The tubes were filled with a hydrogel formulation that can sustain drug release after 3 weeks (**right**). The drugs were metoprolol (METO) and nadolol (NAD); small, tube, and large denote different PLA tube sizes. Reproduced from [202] with permission.

A notable work was recently conducted by modulating sustained drug release from nanocellulose hydrogels by adjusting the inner geometry of implantable capsules [202]. The authors 3D printed capsules using PLA and inserted drug-loaded nanocellulose hydrogels inside them, so that the PLA would sustain the release from the nanofiber hydrogel as shown in Figure 10.

An important work on the fabrication and application of nanofiber hydrogels is worth mentioning here [203]. Therein, the authors reviewed the design and synthesis of nanofibers, as well as their self-assembly into supramolecular hydrogels that originate from small bioactive molecules. They demonstrated their efficient utility in applications such as molecular recognition, wound healing, toxin removal, and drug release. Their review article also described the use of enzymes as switches to modulate the self-assembly of small molecules for nanofiber generation and subsequent hydrogel formation. As mentioned earlier, their review was considered to be a highly motivating work for recent advances in supramolecular hydrogels for sustained drug release [203]. Table 5 lists a number of published sustained drug-release studies conducted by using self-gelling or composite systems carrying different drugs tailored for biomedical applications ranging from cancer treatment to wound healing. Interestingly, the maximum release period for all the systems presented in Table 5 was at least 1 day and in many cases extended into a week. This shows that most nanofiber hydrogels are designed for sustained release, indeed, and they can prolong the release for up to 2 months [204]. The table also revealed that peptides, silk (natural proteins), and cellulose and chitosan (i.e., polysaccharides) are highly popular as nanofiber materials. For composite systems, alginates and polyvinyl alcohol were actively used as the encapsulating gel.

**Figure 11:**
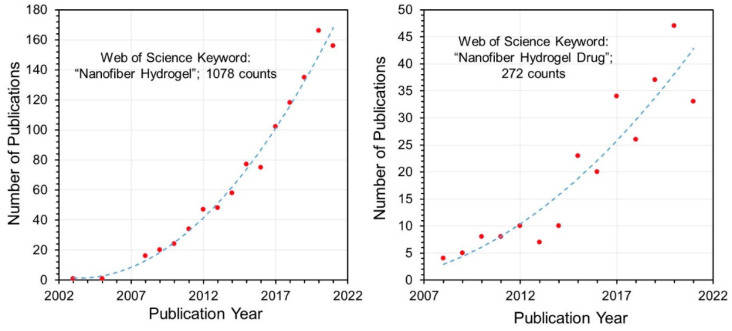
Web of Science keyword search results on “nanofiber hydrogel” and “nanofiber hydrogel drug” searches conducted on 18 October 2021. Number of publications as a function of publication year are plotted for both keywords. Both trends show a second-order polynomial increase.

Using the Web of ScienceTM database with key words such as “Nanofiber Hydrogel” and “Nanofiber Hydrogel Drug”, it was possible to analyze the distribution of publications over the years from 2008 onwards. Figure 11 shows a polynomial increase in the number of publications on nanofiber hydrogel materials. About 25% of the publications use “drug” or “drug release” in their titles, indicating that a significant portion of nanofiber hydrogel research is focused on drug release.

Finally in closing, nanofiber networks can also be fabricated in core-shell format, and these fibers can be very effective as controlled drug delivery vehicles [229]. Furthermore, nanofibers can be fabricated as 3D elastic scaffolds with self-fitting capability and tailorable gradient formation from inorganic materials [230] that can be used for hard tissue generation. The combination of this new generation of electrospun materials with hydrogels is expected to occur soon, and such new nanofiber hydrogels will deliver new advances in biomedical materials science.

## 5. Conclusions

In this review, we showed that the majority of the published works on nanofiber hydrogels could maintain exceptional drug release rates exceeding a few days and even extending into months, which is an extremely difficult task to achieve in the absence of nanofibers. This review also established that some publications still lack vigilant rheological studies on nanofiber hydrogels, although the rheological properties of hydrogels can affect cell function, mechano-transduction, and cellular interactions such as growth, migration, adhesion, proliferation, differentiation, and morphology. This can even be more precarious for 3D or 4D printable gel systems while maintaining efficient drug delivery rates. Polymer nanofibers have extraordinarily high surface areas. This increases the area over which materials can be transported into and out of a system via diffusion and active transport. Similarly, since hydrogels retain a degree of flexibility very similar to natural tissue, due to their substantial water content, hydrogels made from natural or biodegradable macromolecular systems can even be injectable and kept in syringes. Hydrogel transport properties can be easily modified and tailored and can encapsulate nanofiber networks. It has been shown that certain nanofiber networks can be transformed into hydrogels directly, without the need for a hydrogel inclusion. This review compiled such recent advances in the fabrication and application of biomedical nanofiber hydrogels, with special attention to drug release performance. Most of the drug release studies and recent advances have focused on designing self-gelling nanofiber systems made from peptides or other natural proteins loaded with cancer drugs. Next in line are the polysaccharide nanofiber gels that are being developed and studied for drug release. Thirdly, electrospun biodegradable polymer networks embedded in polysaccharide-based hydrogels have been exceedingly popular. There appears to be no clear advantage of one system over the other. However, this will be determined by the practicality of the fabrication method, cost-effectiveness of the materials used, the longevity and controllability of the drug release action, and protection of the drug functionality over a long period within the nanofiber hydrogels.

## Figures and Tables

**Table 1:** Number of reviews and publication periods on different hydrogel applications in biomedical field.

Hydrogel Application	Number of Reviews	Publication Period	Reference
Tissue engineering	4	2011–2020	[81,82,83,84]
Drug delivery	6	1993–2021	[85,86,87,88,89,90]
Wound dressing	6	2017–2020	[91,92,93,94,95,96]
Contact lenses	7	2017–2021	[97,98,99,100,101,102,103]
Bio-sensors	5	2012–2020	[104,105,106,107,108]

**Table 2:** A summary of nanofibers in hydrogels, including types of polymers or polymer blends used and the resultant mechanical properties.

Polymer Type	Spinning Conditions	Mechanical Properties	Biological Additives	Biomedical Applications	Reference
Poly(ε-caprolactone)-hyaluronic acid	Mechanical mixing of spun fibers	Storage moduli similar to skin	n/a	Soft tissue restoration	[131]
Supramolecular peptide	Naturally nanofibrous	Good viscoelastic properties	n/a	Cell delivery	[132]
Collagen/cellulose	Acetic acid	Good compressive strength	n/a	Tissue scaffold	[133]
Polyvinyl alcohol/alginate	Water solution	Good balance between strength and elasticity	n/a	Burn treatment	[134]
Poly(vinyl alcohol)/β-cyclodextrin	PBS solution with different pH levels	Close to 100% elongation	Au nanoparticles	Glucose sensor	[135]
Thiolated hyaluronic acid/polyethylene glycol diacrylate/polycaprolactone	Mixed solvent	Storage moduli similar to spinal cord nervous tissue	n/a	Spinal cord regeneration	[136]
Polycaprolactone/alginate	Mixed solvent	Strength/elasticity similar to peripheral nerve	n/a	Peripheral nerve regeneration	[137]
Polycaprolactone/chondrocyte/alginate	Mixed solvent	Close to 100% elongation	Dexamethasone (Dex)	Cartilage injury	[138]
Fibrin/poly(ethylene oxide)	Saline solution	n/a	Chitosan	Peripheral nerve regeneration	[139]
PuraMatrix^TM^ Peptide Hydrogel	Acidic solution	n/a	Insulin	Subcutaneous injection	[140]
Poly(lactic-co-glycolic acid)/hydroxybutyl chitosan	Mixed solvent	High elastic modulus better than chitosan	n/a	Cartilage tissue engineering, 3D printing	[141]
Chitin	Mixed solvent	n/a	Nanoscale bone minerals	Bone regeneration	[142]
Chitin	Acidic solution	Storage moduli similar to cellulose nanofibers	Silver nanoparticles	Refractory wound management	[143]
Keratin/polyurethane	Acidic solution	Close to 200% elongation	n/a	Tissue engineering gel	[144]
Polycaprolactone-polyethylene glycol (PCL-PEG)	Mixed solvent	n/a	Nucleic acids	Axon regeneration	[145]

**Table 3:** Rheological studies on nanofiber hydrogels are extremely important for effective 3D and 4D printing applications. Oscillatory shear measurements as well as standard temperature dependence measurements are needed for thixotropic gel printing.

Nanofiber Hydrogel	Oscillatory Shear Measurement	Standard Shear Measurement	Temperature Dependence Measurement	Production Method	Targeted Application	Reference
Agarose/acrylamide	Yes	Yes	Yes	4D printing	Scaffolds, soft medical robots	[152]
Poly(vinyl alcohol)/β-cyclodextrin	No	Yes	No	Syringe injection	Glucose sensor	[135]
Cellulose nanofiber	Yes	Yes	no	3D printing	Scaffold, drug release	[153]
Biphenyl-tripeptide	Yes	Yes	No	Solution gelation	Regenerative medicine	[154]
Spider silk	Yes	Yes	No	Solution gelation	Scaffold, drug release	[155]
Liposome-Peptide Nanofiber	Yes	Yes	No	Solution gelation	Regenerative medicine	[156]
Betamethasone phosphate	No	Yes	No	Solution gelation	Tissue injection	[157]
Collagen type I	Yes	Yes	Yes	Self-assembly	Regenerative medicine and drug delivery	[158]
Chitosan/acrylamide	Yes	Yes	No	Radical polymerization	Implants	[159]
Cellulose/polyvinyl alcohol/sodium alginate	No	Yes	No	3D Printing	Scaffold	[160]
Peptide/gelatin	Yes	Yes	Yes	3D Printing	Vascularization	[161]
Bacterial cellulose/silk	Yes	Yes	Yes	3D Printing	Soft tissue repair	[162]
Bacterial cellulose/silk	Yes	Yes	No	3D Printing	Lung tissue regeneration	[163]
Hydroxybutyl chitosan/polycaprolactone	Yes	Yes	No	3D Printing	Cartilage differentiation	[141]
Alginate/gelatin/carbon nanofibers	Yes	Yes	No	3D Printing	Cardiac and neuronal tissue engineering	[164]
Alginate/polylactic acid	Yes	Yes	No	3D Printing	Cardiovascular stents	[165]
Poly(lactide-co-caprolactone)/collagen	No	Yes	No	3D Printing	Nerve regeneration	[166]
Alginate/gelatin/bacterial nanocellulose	Yes	Yes	Yes	3D Printing	Neural tissue engineering	[167]

**Table 4:** A summary of stimuli responsive drug release studies of hydrogels in general.

Type of Study	Hydrogel	Model Drugs	Release Mechanism	Reference
Experimental	Poly(L-lactide)-co-polyethyleneglycol-co-poly(L-lactide) dimethacrylates	Doxorubicin and tetracycline	pH-induced	[175]
Experimental	Poly(*N*-isopropylacrylamide)	Bovine serum albumin	Thermal-induced	[176]
Experimental	low molecular weight gelators (LMWGs)	6-aminoquinoline (6-AQ)	Enzyme-induced	[177]
Experimental	Poly(*N*-isopropylacrylamide)	Vitamin B_12_ and methylene blue	Magnetic-induced	[178]
Experimental	Dextran	Dexamethasone and indomethacin	Electro-induced	[179]
Experimental	Polyacrylic acid/poly(vinyl alcohol)	Indomethacin	Electro-induced	[180]
Review	Several	Several	Electro-induced	[181]
Review	Several	Several	Electro-induced	[182]
Experimental	Low molecular weight gelators (LMWGs)	Doxorubicin	Light-induced	[183]
Experimental	Low molecular weight gelators (LMWGs)	Naproxen, diclofenac, ciprofloxacin, actinomycin D, cytochrome	Light-induced	[184]
Review	Several	Several	Light-induced	[185]
Experimental	Poly([6-bromo-7-hydroxycoumarin-4-yl]methyl methacrylate)	None	Light-induced	[186]
Experimental	Poly(*N*-isopropylacrylamide)/ poly(ethyl acrylate)	Daidzein	pH-induced	[187]
Experimental	Carboxymethyl chitosan/poloxamer	Nepafenac	pH-induced	[188]
Theoretical/experimental	Polyelectrolyte hydrogels	None	pH-induced	[189]
Review	Several	Several	pH-induced	[190]
Experimental	Gelatin	Vitamin B_12_	Magnetic-induced	[191]
Experimental	Chitosan	Curcumin	Magnetic-induced	[192]
Experimental	Chitosan	Adriamycin, rifampicin	Magnetic-induced	[193]
Experimental	Poly(acrylamide–gelatin)	Doxorubicin	Magnetic-induced	[194]
Experimental	Experimental	FITC-dextran	Magnetic-induced	[195]
Review	Several	Several	pH-induced	[196]
Review	Several	Several	Electro-induced	[197]

**Table 5:** Sustained drug release studies of nanofiber hydrogels.

Nanofiber Material	Gelation Process	Composite System	Composite Components	Type of Drug	Release Medium	Maximum Release Period (Day)	Reference
Chitosan-graft-poly(N-isopropylacrylamide) (CTS-g-PNIPAAm)	Swelling	Yes	PEO	BSA	HCl-KCl pH 2.2; PBS, pH 7.4	2.5	[203]
Polydopamine-intercalated silicate/	Gelling in solution	Yes	Chitosan/gelatin	Tetracycline hydrochloride	PBS, pH 7.4	5–6	[205]
Polyaniline	Gelling in solution	Yes	Polyacrylamide	Amoxicillin	PBS, pH 7.4	0.21	[206]
Silk	Gelling in solution	No	n/a	Doxorubicin	PBS pH 4.5, 6.0, or 7.4	21–56	[204]
Silk	Gelling in solution/thermal	No	n/a	Desferrioxamine	PBS, pH 7.4	40	[207]
Peptide	Gelling/self-assembling in solution	No	n/a	Timolol Maleate	PBS, pH 7.4	1	[208]
Peptide	Gelling/self-assembling in solution	No	n/a	Human antibodies	PBS, pH 7.4	12	[209]
Peptide	Gelling/self-assembling in solution	No	n/a	Recombinant receptor activator of NF-kB ligand	PBS, pH 7.4	4	[210]
Peptide amphiphiles	Gelling/self-assembling in solution	No	n/a	Doxorubicin	PBS, pH 7.4	7	[211]
Coiled-coil protein, Q,	Gelling/self-assembling in solution	No	n/a	Curcumin	DMSO buffer	18	[212]
Peptide	Gelling/self-assembling in solution	No	n/a	Lysozyme, trypsin inhibitor, BSA, IgG	PBS, pH 7.4	2–3	[213]
Peptide (RADA 16)	Gelling/self-assembling in solution	No	n/a	Pindolol, Quinine and Timolol	PBS, pH 7.4	7	[214]
Cellulose	Cross-linking in solution	Yes	Polyvinyl alcohol	Cisplatin	PBS, pH 7.4	18	[215]
Silk	Gelling in solution/thermal	Yes	Concentrated mesenchymal stem cells	Various growth factors	PBS, pH 7.4	2	[216]
d-Amino Acid Dipeptide	Gelling/self-assembling in solution	No	n/a	Folic acid	HEPES buffer	0.2–1	[217]
Bacterial cellulose	Cross-linking in solution	Yes	Sodium alginate	Ibuprofen	PBS, pH 7.4 and other pHs	1.5	[218]
Cellulose	Commercial	No	n/a	FITC-dextrans, d-(+)-trehalose dehydrate, Metronidazole, Nadolol, Ketoprofen, Lysozyme	PBS, pH 7.4	6	[219]
Peptide hydrosol	Gelling/self-assembling in solution	No	n/a	Insulin	PBS, pH 7.4	0.5	[140]
Fibrin	Gelling in solution	Yes	Poly(DL-lactic-co-glycolic acid)	Albumin from bovine Serum, FITC conjugate	PBS, pH 7.4	83	[220]
Polycaprolactone	Cross-linking in solution	Yes	Alginate	Adenosine triphosphate	PBS, pH 7.4	12	[221]
Peptide	Gelling/self-assembling in solution	No	n/a	Olsalazine	Buffer, pH 5	n/a	[222]
Poly(vinyl alcohol-co-ethylene)	Cross-linking in solution	Yes	Alginate	Vancomycin	PBS, pH 7.4	5	[223]
Peptide amphiphile	Gelling/self-assembling in solution	No	n/a	Prodan	PBS, pH 7.4-DMSO	40	[224]
Polydopamine	Gelling/self-assembling in solution	Yes	Alginate	Bortezomib	PBS, pH 5–7.4	7	[225]
Peptide	Gelling/self-assembling in solution	No	n/a	BSA-linked cisplatin	PBS, pH 7.4	1	[226]
*N*-(9-fluorenylmethoxycarbonyl)-L-phenylalanine	Gelling/self-assembling in solution	Yes	Puerarin	Berberine hydrochloride	PBS, pH 5.8	1.5	[227]
Betamethasone phosphate	Gelling/self-assembling in solution	No	No	PDL1 antibody	PBS, pH 7.4	7	[157]
Polyvinylpyrrolidone	Gelling/self-assembling in solution	No	No	Hydroxycinnamic acids	PBS, pH 7.4	9	[228]

## Data Availability

Not applicable.
